# A multicentre prospective observational study comparing arterial blood gas values to those obtained by pulse oximeters used in adult patients attending Australian and New Zealand hospitals

**DOI:** 10.1186/s12890-019-1007-3

**Published:** 2020-01-09

**Authors:** Janine Pilcher, Laura Ploen, Steve McKinstry, George Bardsley, Jimmy Chien, Lesley Howard, Sharon Lee, Lutz Beckert, Maureen Swanney, Mark Weatherall, Richard Beasley

**Affiliations:** 1 Medical Research Institute of New Zealand, Private Bag 7902, Wellington, 6242 New Zealand; 2 Capital & Coast District Health Board, Wellington, New Zealand; 3 Victoria University of Wellington, Wellington, New Zealand; 4 Canterbury District Health Board, Christchurch, New Zealand; 5 Department of Respiratory and Sleep Medicine, Ludwig Engel Centre for Respiratory Research, University of Sydney at Westmead Hospital, Sydney, Australia; 6 University of Otago Wellington, Wellington, New Zealand

**Keywords:** Arterial blood gas, Hypoxaemia, Oxygen, Pulse oximeter, Validation

## Abstract

**Background:**

Pulse oximetry is widely used in the clinical setting. The purpose of this validation study was to investigate the level of agreement between oxygen saturations measured by pulse oximeter (SpO_2_) and arterial blood gas (SaO_2_) in a range of oximeters in clinical use in Australia and New Zealand.

**Methods:**

Paired SpO_2_ and SaO_2_ measurements were collected from 400 patients in one Australian and two New Zealand hospitals. The ages of the patients ranged from 18 to 95 years. Bias and limits of agreement were estimated. Sensitivity and specificity for detecting hypoxaemia, defined as SaO_2_ < 90%, were also estimated.

**Results:**

The majority of participants were recruited from the Outpatient, Ward or High Dependency Unit setting. Bias, oximeter-measured minus arterial blood gas-measured oxygen saturation, was − 1.2%, with limits of agreement − 4.4 to 2.0%. SpO_2_ was at least 4% lower than SaO_2_ for 10 (2.5%) of the participants and SpO_2_ was at least 4% higher than the SaO_2_ in 3 (0.8%) of the participants. None of the participants with a SpO_2_ ≥ 92% were hypoxaemic, defined as SaO_2_ < 90%. There were no clinically significant differences in oximetry accuracy in relation to clinical characteristics or oximeter brand.

**Conclusions:**

In the majority of the participants, pulse oximetry was an accurate method to assess SaO_2_ and had good performance in detecting hypoxaemia. However, in a small proportion of participants, differences between SaO_2_ and SpO_2_ could have clinical relevance in terms of patient monitoring and management. A SpO_2_ ≥ 92% indicates that hypoxaemia, defined as a SaO_2_ < 90%, is not present.

**Trial registration:**

Australian and New Zealand Clinical Trials Registry (ACTRN12614001257651). Date of registration: 2/12/2014.

## Background

Pulse oximeter measured oxygen saturation is a non-invasive approximation of arterial oxygen saturation (SpO_2_), which is considered the fifth vital sign in clinical assessment [1–3]. In clinical practice monitoring of SpO_2_ values is required to titrate oxygen therapy to avoid the risks of hypoxaemia and hyperoxaemia [1, 2].

Assessment of agreement between the gold standard arterial blood gas (ABG) measurement of oxygen saturation (SaO_2_) and SpO_2_ is essential for the interpretation and use of pulse oximetry values. It is also essential for the development of safe and practical recommendations for SpO_2_ targets for the titration of oxygen therapy. Overestimation of actual SaO_2_ may mean clinically relevant hypoxaemia is not detected or treated. Conversely, underestimation of actual SaO_2_ may result in unnecessary oxygen therapy with the associated risks of hyperoxaemia.

The United States regulatory body, the Food and Drug Administration (FDA) centre, requires the accuracy of pulse oximeters to be tested against SaO_2_, in healthy adults in laboratory settings [4]. In clinical practice a number of factors influence oximeter accuracy including the degree of hypoxaemia, hypercapnia, glycosylated haemoglobin (HbA1c), skin pigmentation, movement artefacts, peripheral perfusion and use of nail polish or acrylic nails [3, 5–12]. Clinical studies report that SpO_2_ can both over and underestimate SaO_2,_ and the values may have wide limits of agreement [5–32]. However, oximeter accuracy may also differ by oximeter model [7, 8, 12, 18, 19]. Manufacturers are continuously evolving sensor technology and software algorithms [3]. This means previous studies may not be directly relevant to current clinical practice because of the population groups and oximeter models used.

In our recent study investigating the accuracy of oximeters used in Australian and New Zealand Intensive Care Units (ICUs), we demonstrated a mean bias for SaO_2_ minus SpO_2_ of only 0.15%, with limits of agreement plus or minus 4.4% [18]. In this study we aim to investigate the agreement between SaO_2_ and SpO_2_ measurements by oximeters currently in use in Australian and New Zealand hospitals outside the critical care setting, either on the ward or in the Emergency (ED), High dependency Unit (HDU) or outpatient departments. Secondary objectives were to evaluate the diagnostic performance of SpO_2_ to detect hypoxaemia, and investigate factors affecting oximeter accuracy.

## Methods

This multicentre prospective non-experimental observational study compared simultaneous SpO_2_ and SaO_2_ measurements in inpatients and outpatients at Westmead Hospital in Australia, and Wellington and Christchurch Hospitals in New Zealand. It was prospectively registered on the Australian and New Zealand Clinical Trials Registry (ACTRN12614001257651). Ethical approval was obtained from the Northern B Ethics Committee in New Zealand (14/NTB/115) and the Western Sydney Local Health District Human Research Ethics Committee in Australia (LNR/14/WMEAD/387).

Patients aged 16 years or older who were to have an ABG measurement as part of routine clinical care were recruited. Full written informed consent was provided in New Zealand by participants, or next of kin if participants were unable to (for example, if they were too unwell). Participants were not recruited if they had a diagnosis of sickle cell anaemia, methaemoglobinemia, carbon monoxide (CO) poisoning, or were previously recruited to the study and had paired SpO_2_ and SaO_2_ values successfully recorded. They could also be excluded for any other condition which, at the investigator’s discretion, was believed may present a safety risk or impact upon the feasibility of the study or the interpretation of the study results.

Participants were identified in hospital wards and outpatient clinics. Demographic data were recorded. Skin colour was assessed using the Fitzpatrick scale [33].

SpO_2_ was measured during a clinically indicated ABG. The oximeter probe was put in place for at least 10 s prior to the ABG, or longer if indicated by manufacturer’s instructions. SpO_2_ was measured from an earlobe or finger probe, depending on departmental policies and what the staff member responsible for performing oximetry would usually use to monitor that patient. If a finger probe was used it was placed on the index finger on the contra-lateral side to ABG sampling. Where possible, nail polish was removed before measurement.

The SpO_2_ value recorded was the value on the oximeter when blood was first observed to enter the ABG collection vial. If the participant was receiving supplementary oxygen at the time of the ABG, this was also recorded. Measurements paired with ABG samples subsequently identified to be venous or unusable, e.g. sample too small for analysis, were excluded. The models of oximeter and ABG analyser were recorded. Data recorded from the ABG were SaO_2_, partial pressure of oxygen (PaO_2_), partial pressure of carbon dioxide (PaCO_2_), Carboxyhaemoglobin (CoHb), Methaemoglobin (MetHb) and HbA1c, if measured as part of clinical practice. Investigators were asked to record whether they had any concerns with oximeter accuracy, such as nail polish that was not removed, poor oximeter signal, or patient movement. Participants in which there was a reported concern with oximeter accuracy were not excluded from analyses.

Bland Altman plots and estimation of bias and limits of agreement were used to describe the agreement between SpO_2_ and SaO_2_ measurement, using SaO_2_ as the reference standard.

The diagnostic performance of SpO_2_ < 90% to detect hypoxaemia, defined as a SaO_2_ < 90% and defined as a PaO_2_ < 60 mmHg, was evaluated using contingency tables, with sensitivities and specificities estimated by an exact binomial method for proportions. A post hoc analysis of the ability for SaO_2_ < 90% to detect a PaO_2_ < 60 mmHg was performed using the same methods.

Associations with mean bias were illustrated by a scatter plot with a scatter plot smoother and a Spearman rank-correlation coefficient for SaO_2_, and ANOVA for categorical variables in Table 1. The mean difference between categories was assessed with an F-test. Where a categorical variable only had one observation it was not used in the ANOVA. If important predictors of bias were identified, it was planned to use Bland Altman methods determine whether there was also an effect on limits of agreement.

**Table 1:** Categorical factors assessed for influence on oximeter accuracy

Location of measurement (ED, HDU, ward, or outpatient department)	
Position of the oximeter probe (finger or ear)	
Recognised condition associated with chronic respiratory failure (chronic obstructive pulmonary disease, obesity hypoventilation syndrome, bronchiectasis, cystic fibrosis, neuromuscular disease and chest wall deformities such as severe kyphoscoliosis)	
Current tobacco smoking status (current versus ex or non-smoker)	
Skin pigmentation (based on modified Fitzpatrick scale with patient skin colour classified as either: Light (Type I to Type II), Medium (Type III to Type IV) or Dark (Type V to Type VI))	
Diabetes Mellitus	

*ED* emergency department, *HDU* high dependency unit

To estimate the difference between SpO_2_ and SaO_2_ due to different oximetry devices, estimation of variance components and associated intra-class correlation coefficients for the effect of oximeters as well as best linear unbiased predictors of the effect of individual oximeters were assessed by mixed linear models and estimation by restricted maximum likelihood.

SAS version 9.4 was used.

The planned sample size of 400 was based on three considerations. Firstly, for the analysis of variables that predict the size of the bias we sought to have between 20 and 40 participants for each degree of freedom in the ANOVA. Based on the six variables, some of which have multiple levels, this required between 200 and 400 participants. Secondly the estimates of paired SD for the SpO_2_ to SaO_2_ difference from patients in a range of clinical settings were 0.55% [6], 2.1% [17], and 2.2% [16]. There is 80% power, with a type I error rate of 5%, to detect a SpO_2_ to SaO_2_ difference of 2% for any of the variables that might predict bias, if there were two equal sized groups of 21 participants. For estimation of variance of components for the different pulse oximeters by Best Unbiased Linear Predictors between 20 and 25 participants per oximeter brand were required and it was estimated that between 10 and 20 oximeter brands would be used.

## Results

### Participants

**Fig. 1:**
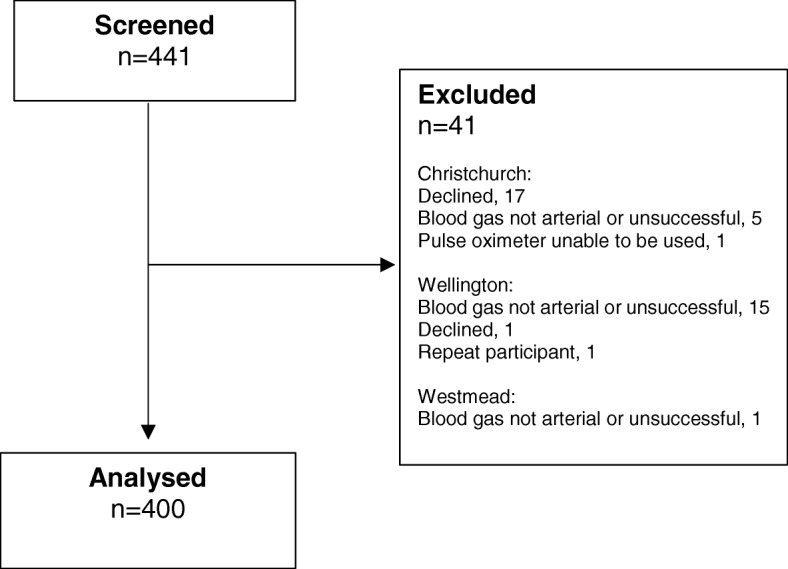
Flow of participants through the study

Four-hundred patients were recruited; 253 from Christchurch, 103 from Wellington and 44 from Westmead Hospital (Fig. 1). Participant characteristics and details of the pulse oximeters and ABG analysers are presented in Table 2.

**Table 2:** Participant characteristics (*N* = 400)*

General characteristics	N (%)*	
Age, years		
Mean (SD)	64.2 (15.2)	
Min to max	18.7 to 95.1	
Male gender	212 (53)	
Smoking status (*n* = 399)		
Current	43 (10.8)	
Ex	203 (50.9)	
Never	153 (38.5)	
Fitzpatrick Score		
I	44 (11)	
II	198 (49.5)	
III	127 (31.8)	
IV	30 (7.5)	
V	1 (0.3)	
VI	0 (0)	
Conditions associated with chronic respiratory failure		
None:	229 (57.3)	
Hypercapnia** on ABG	16	
At least one:	171 (42.8)	
Hypercapnia** on ABG	57	
Individual conditions associated with chronic respiratory failure		
Chronic obstructive pulmonary disease	113 (28.3)	
Obesity hypoventilation syndrome	30 (7.5)	
Bronchiectasis	19 (4.8)	
Cystic fibrosis	1 (0.3)	
Neuromuscular disease	24 (6)	
Chest wall deformity	11 (2.8)	
Peripheral vascular disease	11 (2.8)	
Diabetes	80 (20)	
Oxygen administration	25 (6.3)	
New Zealand Ethnicity		
NZ European	280 (70.0)	
Māori	26 (6.5)	
Samoan	13 (3.3)	
Chinese	1 (0.3)	
Indian	4 (1.0)	
Cook Island Māori	1 (0.3)	
Tongan	3 (0.8)	
Other	28 (7.0)	
Australian Ethnicity		
Caucasian	34 (8.5)	
Middle Eastern	6 (1.5)	
Other	5 (1.3)	
Hospital location		
Outpatient	341 (85.3)	
Ward	40 (10)	
HDU	18 (4.5)	
ED	1 (0.3)	
ABG and oximetry data	Mean (SD), min-max*	Median (IQR)
SpO_2_, %	93.5 (3.8), 72 to 100	94 (92 to 96)
Participants with SpO_2_<90% (N, (%))	49 (12.3)	
Concern with SpO_2_ data accuracy recorded by investigator (N, (%))***	16 (4.0)	
SaO_2_, %	94.7 (3.8), 72.1 to 100	95.7 (93.2 to 97.1)
Participants with SaO_2_<90% (N, (%))	35 (8.8)	
PaO_2_, mmHg	74.8 (21.3), 37.9 to 396	73 (64.7 to 83)
Participants with PaO_2_<60 mmHg (N, (%))	61 (15.3)	
PaCO_2_, mmHg	40.3 (7.2), 25.4 to 87.2	39 (35.8 to 43.4)
Hb, g/L	136.7 (18.8), 67 to 192	137 (126.5 to 149)
CoHb, %		
All participants (*N* = 358)	2.1 (1.2), 0 to 7	1.8 (1.4 to 2.3)
Current smokers (*N* = 40)	3.9 (1.7), 0.9 to 6.7	4.0 (2.4 to 5.4)
Ex smokers (*N* = 183)	1.9 (0.9), 0.0 to 7.0	1.7 (1.4 to 2.2)
Never smokers (*N* = 135)	1.9 (0.7), 0.0 to 4.2	1.7 (1.3 to 2.3)

ABG: Arterial blood gas, CoHb: Carboxyhaemoglobin, COPD: Chronic Obstructive Pulmonary Disease, ED: Emergency Department, Hb: Haemoglobin, HDU: High Dependency Unit, ILD: Interstitial lung disease, NZ: New Zealand, NMD: Neuromuscular disease, OSA/OHS: Obstructive sleep apnoea and/or obesity hypoventilation syndrome, PaCO_2_: Partial pressure of arterial carbon dioxide, PaO_2_: Arterial partial pressure of oxygen, SaO_2_: Oxygen saturation measured by arterial blood gas sample, SpO_2_: Oxygen saturation measured by standard pulse oximeter

*Unless otherwise stated

**PaCO_2_ > 45 mmHg

***For example nail polish, acrylic nail, nail pathology, low perfusion or variability noted on monitor

Further details available in Online Additional File, including oximeter models and ABG analysers used (Additional file 1: Table S1)

### Agreement between SpO_2_ and SaO_2_

**Fig. 2:**
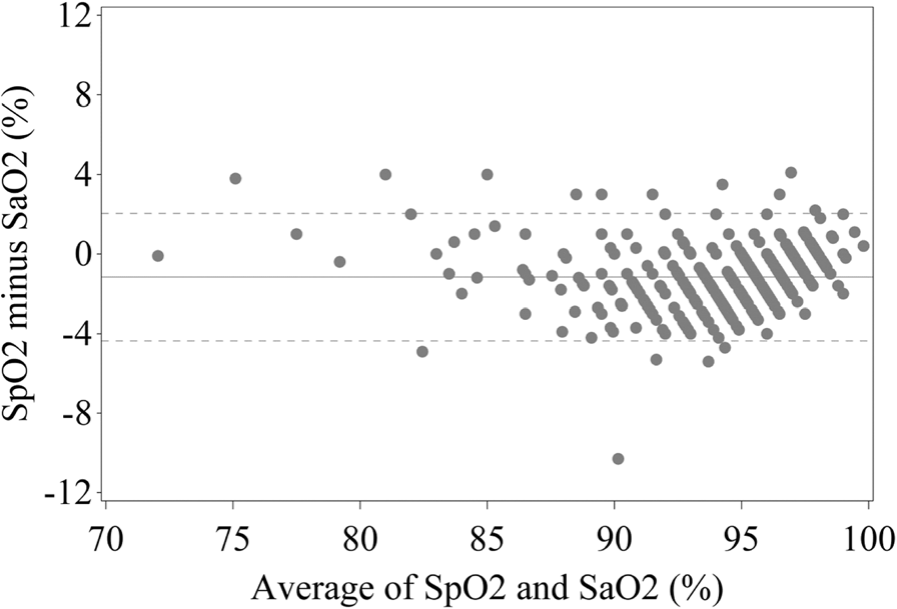
Bland Altman Plot for SpO_2_ versus SaO_2_ SaO_2_: Oxygen saturation measured by arterial blood gas sample, SpO_2_: Oxygen saturation measured by standard pulse oximeter. The solid reference line is the mean bias and the dashed reference lines are the limits of agreement around this mean bias.

The bias for SpO_2_ minus SaO_2_ was − 1.2%, with limits of agreement − 4.4 to 2.0%. The Bland Altman plot is shown in Fig. 2. In 10/400 (2.5%) participants the SpO_2_ was at least 4% lower than SaO_2_. In one of these participants the investigator reported concern with oximeter accuracy. In 3/400 (0.8%) participants the SpO_2_ was at least 4% higher than the SaO_2_. In one of these participants the investigator reported concern with oximeter accuracy. Characteristics of these participants are in the Online Additional file 1: Table S2).

### Detection of hypoxaemia

**Fig. 3:**
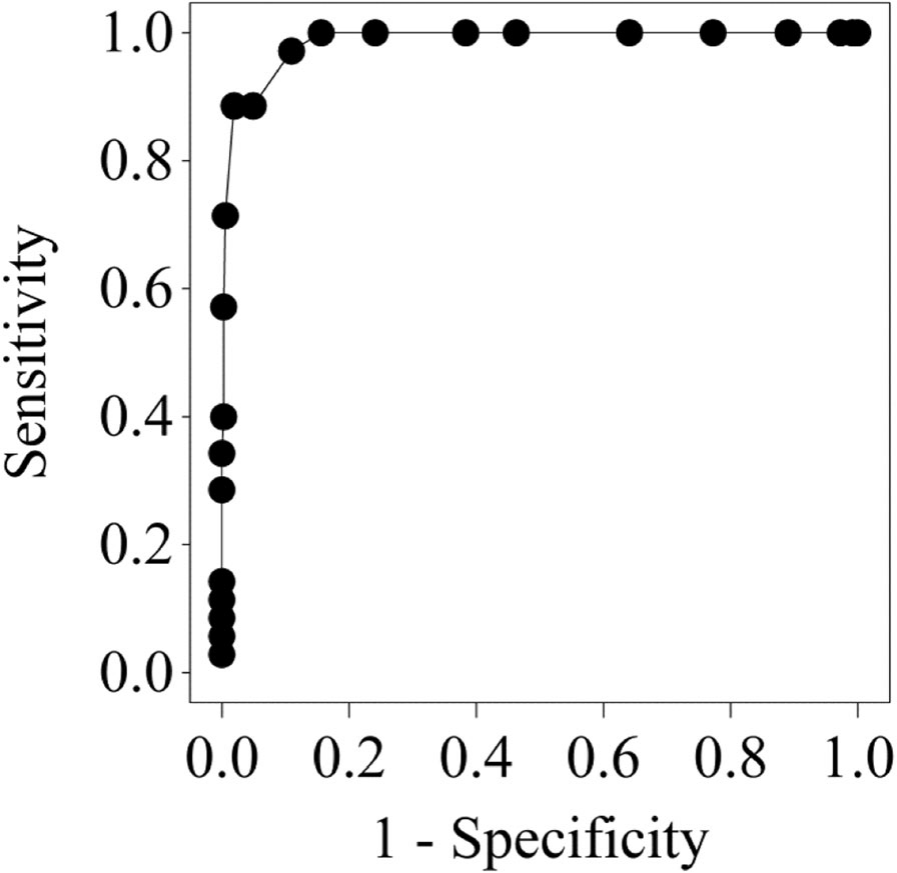
ROC curve for SpO_2_ to predict SaO_2_ < 90%. The c-statistic for the logistic regression, representing the area under the ROC curve, was 0.986. SaO_2_: Oxygen saturation measured by arterial blood gas sample, SpO_2_: Oxygen saturation measured by standard pulse oximeter

Sensitivity and specificity for the ability of SpO_2_ < 90% or < 92% to detect SaO_2_ < 90%, the ability for SpO_2_ < 90% to detect PaO_2_ < 60 mmHg, and the ability for SaO_2_ < 90% to detect PaO_2_ < 60 mmHg, are shown in Table 3. The ROC curve for SpO_2_ to detect SaO_2_ < 90% is shown in Fig. 3. SpO_2_ < 92% had 100% sensitivity and 84.4% specificity for detecting SaO_2_ < 90%, and 95.1% sensitivity and 90.0% specificity for detecting PaO_2_ < 60 mmHg. See the Online Additional File for tabulated values and ROC curve (Additional file 1: Tables S3 and S4, Additional file 1: Figure S1). Participants tended to sit to the left of the predicted oxygen haemoglobin dissociation curve (Online Additional file 1: Figure S2) [34]. In 13/400 (3%) of participants the PaO_2_ was > 100 mmHg. Twelve of these participants had a SpO_2_ > 96%. One had an oximetry value of 96%; their PaO_2_ was 142 mmHg and SaO_2_ was 99%.

**Table 3:** Diagnostic performance of SpO_2_ and SaO_2_

Ability for SpO_2_ <90% to detect SaO_2_ <90%			
	SaO_2_ <90%		Sensitivity: 88.6%
SpO_2_ <90%	Yes	No	Specificity: 95.1%
Yes	31	18	
No	4	347	
Total	35	365	
Ability for SpO_2_ <92% to detect SaO_2_ <90%			
	SaO_2_ <90%		Sensitivity: 100%
SpO_2_ <92%	Yes	No	Specificity: 84.4%
Yes	35	57	
No	0	308	
Total	35	365	
Ability for SpO_2_ <90% to detect PaO_2_ <60 mmHg			
	PaO_2_ <60 mmHg		Sensitivity: 70.5%
SpO_2_ <90%	Yes	No	Specificity: 98.2%
Yes	43	6	
No	18	333	
Total	61	339	
Ability for SaO_2_ <90% to detect PaO_2_ <60 mmHg			
	PaO_2_ <60 mmHg		Sensitivity: 54.1%
SaO_2_ <90%	Yes	No	Specificity: 99.4%
Yes	33	2	
No	28	337	
Total	61	339	

PaO_2_: Partial pressure of oxygen, SaO_2_: Oxygen saturation measured by arterial blood gas sample, SpO_2_: Oxygen saturation measured by standard pulse oximeter

### Factors potentially influencing oximeter accuracy

There was no statistical evidence of an association between SaO_2_ and bias between SpO_2_ and SaO_2_; Spearman coefficient 0.003, *P* = 0.94. Of the other factors from Table 1, only a diagnosis of diabetes was identified as a predictor of bias (*P* = 0.05). In diabetics it was − 0.8 (95% limits of agreement − 4.4 to 2.8), in non-diabetics it was − 1.2 (− 4.4 to 2.0). Detailed results are presented in the Online Additional File (Additional file 1: Figure S3 and Table S5).

There were at least 14 different oximeter models used. The most common oximeter models used were the Nonin Avant 9700 in 103 participants (26%), Massimo Rainbow Radical 7 in 92 participants (23%) and the Nonin Avant 4000 in 76 participants (19%) (See Additional file 1: Table S1 for all models). The difference in the estimation of variance components was 0.16 for oximeter brand and 2.48 for residual, resulting in an intra-class correlation coefficient of 0.94. This can be interpreted as approximately 6% of variation in the relationship between SpO_2_ versus SaO_2_ being due to oximeter brand. Detailed results by oximeter are shown in the Online Additional file 1: Table S6).

Concern with oximeter accuracy was reported by investigators in 16 patients, nine of which had nail polish, acrylic nail or double nail. Other causes for concern are presented in the Online Additional file 1: Table S1).

## Discussion

The bias and limits of agreement between SpO_2_ and SaO_2_ suggest that pulse oximetry is an accurate method to assess SaO_2_ in most adult patients in the clinical setting. However, in a small number of participants potentially clinically important differences between SpO_2_ and SaO_2_ could affect patient assessment and management. A practical guide that can be derived from these data is that a SpO_2_ ≥ 92% effectively rules out presence of hypoxaemia, indicated by a SaO_2_ < 90%. There were no clinically significant differences in oximeter accuracy based on absolute level of SaO_2_, hospital location, numerous clinical characteristics or oximeter brand.

The magnitude of bias and associated limits of agreement from the range of oximeters in this study suggested that overall they perform at a similar level or better than oximeters used in many of the clinical studies performed in the last 10 years [5, 6, 8, 10–12, 18–26, 28, 30, 31]. This is in keeping with constant oximeter sensor technology and software improvements by manufacturers over time [3]. Specifically, the bias and limits of agreement for SaO_2_ minus SpO_2_ were similar to the values recently obtained in critically unwell patients in the ICU setting (0.15%, limits of agreement plus or minus 4.4%) [18].

The negative bias of − 1.2%, albeit small, meant that the oximeters tended to underestimate SaO_2_. Such underestimation has the potential to result in a conservative estimate of risk of hypoxaemia and may lead to more liberal oxygen therapy than required. SpO_2_ underestimated SaO_2_ by at least 4% in around 3% of participants, and overestimated it by at least 4% in less than 1% of participants. These findings mean that while the oximeters performed well overall, there were still potentially clinically relevant differences in SpO_2_ and SaO_2_ in a small proportion of the participants. In the majority of the participants with SpO_2_ and SaO_2_ values differing by at least 4% the investigators did not state they had any concerns with oximeter accuracy. This highlights the potential difficulty in identifying when an oximetry value is incorrect and emphasises the importance of guideline recommendations to consider oximetry values in clinical context [3].

The TSANZ [2] and BTS [1] guidelines for acute oxygen therapy both recommend use of pulse oximetry as a vital sign and tool to titrate oxygen therapy to a target oxygen saturation range. The TSANZ recommend oxygen is delivered to a SpO_2_ target range of 92 to 96% in patients not at risk of hypercapnic respiratory failure [2]. This range was developed to reduce the risks of both hyperoxaemia and hypoxaemia, while recognising potential oximeter accuracy limitations [35]. The lower limit of 92% is supported by a SpO_2_ saturation of ≥92% indicating that hypoxaemia (SaO_2_ < 90%) is not present. The recommended upper SpO_2_ limit of 96%, aimed at avoiding hyperoxaemia, is supported by the finding that 12 of the 13 participants with a PaO_2_ of greater than 100 mmHg had a SpO_2_ value over 96%.

A SpO_2_ < 90% had a specificity of only 70.5% in identifying a PaO_2_ < 60 mmHg, while for SaO_2_ < 90% it was only 54.1%. These values are in keeping with the majority of participants being positioned to the left of the predicted oxygen haemoglobin dissociation curve. In keeping with recommendations by the TSANZ Oximetry Guidelines [3], these findings highlight the limitations of estimating PaO_2_ from saturation values, and vice versa.

Patients with sickle cell anaemia, methaemoglobinemia, or CO poisoning were excluded from the study and nail polish was removed where possible as these factors are well established to impact on oximeter results [3]. SaO_2_, oximeter model and the numerous clinical variables were not found to significantly impact on oximeter accuracy. However, it was not possible to evaluate the effect of earlobe oximetry, Fitzpatrick scale V or VI, or ED location on accuracy due to there being only one participant in each of these categories.

This study had the advantage of a multicentre design and use of a range of oximeters routinely available to clinical staff in a variety of hospital settings. A wide range of adult patients were included, both in terms of presenting diagnosis and illness severity. While there were a range of SaO_2_ values between 72 and 100%, the results cannot be applied to patients with a SaO_2_ of under 70%, at which oximeter inaccuracy is well recognised [3]. Results may not be applicable to paediatric patients or adult patients in theatre, ICU or ED, especially as a variety of factors specific to these patients have been previously identified as affecting oximeter accuracy [11, 15, 17, 25, 27, 28, 30, 31]. Having only one participant with a Fitzpatrick score of V, and none with VI, meant study findings may not be applicable to patients with higher skin pigmentation. This is especially important as oximeter accuracy has been demonstrated to decrease as pigmentation increases, particularly at lower SaO_2_ levels and in oximeters of the same brand as some of those used in our study (Massimo Radical and Nonin 9700) [7].

Single oximeter and ABG measurement pairing from each participant were used, which has the advantage of removing potential bias from repeated measures in the same participant. However, this did mean we could not specifically assess the accuracy of SpO_2_ to detect changes in SaO_2_ over time.

## Conclusions

Overall, the oximeters in this study had good accuracy in determining individual SaO_2_ values and detecting hypoxaemia in a range of clinical settings. The use of a SpO_2_ of 92% as the lower boundary for the titration of oxygen therapy was supported by 100% sensitivity for SpO_2_ < 92% in identifying hypoxaemia (SaO_2_ < 90%). In a small number of participants discrepancies between SpO_2_ and SaO_2_ could have implications for patient assessment and management. This highlights the importance interpreting SpO_2_ within clinical context.

## Supplementary information

**Additional file 1.** This is an online supplement containing additional details and data as per the manuscript text.

## Data Availability

The datasets used and/or analysed during the current study are available from the corresponding author on reasonable request.

## Abbreviations

ABG: Arterial blood gas
BTS: British Thoracic Society
CoHb: Carboxyhaemoglobin
ED: Emergency department
HbA1c: Haemoglobin A1c
HDU: High dependency unit
ICU: Intensive Care Unit
MetHb: Methaemoglobin
PaCO_2_: Partial pressure of arterial carbon dioxide
Pulse Oximeter: Pulse oximeter used to detect oxygen saturation
SaO_2_: Oxygen saturation measured by arterial blood gas sample
SpO_2_: Oxygen saturation measured by pulse oximetry
TSANZ: Thoracic Society of Australia and New Zealand
