# Supplementary material for: A multicentre prospective observational study comparing arterial blood gas values to those obtained by pulse oximeters used in adult patients attending Australian and New Zealand hospitals
Source: BMC Pulm Med. 2020 Jan 9;20:7. doi: 10.1186/s12890-019-1007-3 (PMC6953261; doi:10.1186/s12890-019-1007-3)
Supplement: Supplementary file 1 — Additional file 1. This is an online supplement containing additional details and data as per the manuscript text. [file 12890_2019_1007_MOESM1_ESM.docx]

**A MULTICENTRE STUDY COMPARING ARTERIAL BLOOD GAS VALUES TO THOSE OBTAINED BY PULSE OXIMETERS USED IN ADULT PATIENTS ATTENDING AUSTRALIAN AND NEW ZEALAND HOSPITALS: SUPPLEMENTAL MATERIAL**

Janine Pilcher,^1,2,3^ Laura Ploen,^4^ Steve McKinstry,^1^ George Bardsley,^1,2^ Jimmy Chien,^5^ Lesley Howard,^5^ Sharon Lee,^5^ Lutz Beckert,^4^ Maureen Swanney,^4^ Mark Weatherall,^6^ Richard Beasley^1,2^

^1^Medical Research Institute of New Zealand, Wellington

^2^Capital & Coast District Health Board, Wellington

^3^Victoria University, Wellington

^4^Canterbury District Health Board

^5^Department of Respiratory and Sleep Medicine, Ludwig Engel Centre for Respiratory Research, University of Sydney at Westmead Hospital

^6^University of Otago, Dunedin

**Contact:**

Professor Richard Beasley, Medical Research Institute of New Zealand

Private Bag 7902, Wellington, New Zealand

Telephone: +64-4-805 0147, Facsimile: +64-4-472 9224

Email: Richard.beasley@mrinz.ac.nz

**Table S1 Oximeter and ABG analyser details**

|  | Christchurch Hospital  (n=253) | Wellington Hospital  (n=103) | Westmead Hospital  (n=44) |
| --- | --- | --- | --- |
| **Oximeter brand** (N, %) | | | |
| **Nonin** | | | |
| Avant 9700 | 103 (40.9) |  |  |
| Avant 4000 | 76 (30.2) |  |  |
| Avant Unspecified* | 1 (0.4) |  |  |
| Lifesense Medair | 48 (19) |  |  |
| 2120 |  | 8 (7.8) |  |
| 2140 |  | 1 (1) |  |
| **Masimo** | | | |
| Masimoset Quartz Q400 | 23 (9.1) |  |  |
| Masimoset Quartz Unspecified* | 1 (0.4) |  |  |
| Rainbow Radical 7 |  | 91 (88.4) | 1 (2.3) |
| **Other** | | | |
| Novametrix Model 512 |  | 1 (1) |  |
| GE Dash 3000 |  | 2 (1.9) |  |
| Welch Allyn monitors** with a Nellcor probe |  |  | 19 (43.2) |
| Philips Intellivue MP70 monitor with a GE TruSignal, Nellcor or Philips probe |  |  | 16 (36.4) |
| Ohmeda Biox 3700E monitor with a GE TruSignal or Nellcor probe |  |  | 7 (15.9) |
| Carescape Monitor B450 with a Nellcor probe |  |  | 1 (2.3) |
| **Concerns with SpO_2_ data accuracy as recorded by the investigator** (N) | | | |
| Nail polish, acrylic nail or double nail | 4 | 4 | 1 |
| Variability in displayed SpO_2_ | 1 | 0 | 2 |
| Low perfusion noted on SpO_2_ monitor | 0 | 1 | 0 |
| Other | 1* | 1*** | 1* |
| **ABG analyser** | Radiometer ABL800 | Radiometer ABL800 | Radiometer ABL800 |

ABG: Arterial blood gas, SpO_2_: Oxygen saturation measured by standard pulse oximeter.

*Unspecified by investigator. **Welch Allyn monitors were either Welch Allyn 300 series, Welch Allyn 52000 series, Welch Allyn Spot Vital Signs LXi with n values for each type not available. *** SpO_2_ probe had to be applied to same hand as ABG taken from due to medical condition affecting contralateral side. Note oximeter monitor type not available for 1 participant from Christchurch.

**Table S2 Participants with discrepancy of at least 4% between SpO_2_ and SaO_2_**

|  | **Condition with Hypercapnia risk** | **Hospital and location** | **Presenting diagnosis** | **Oxygen delivery*** | **Oximeter model** | **PaO_2_**  **_(mmHg)_** | **SaO_2_**  **_(%)_** | **SpO_2_**  **_(%)_** | **SpO_2_ minus SaO_2 (%)_** |
| --- | --- | --- | --- | --- | --- | --- | --- | --- | --- |
| 1 | None | Wellington Outpatients | Interstitial lung disease | No | Masimo Radical 7 | 65 | 94 | 90 | -4 |
| 2 |  | Christchurch Outpatients | Work up | No | Nonin Avant 9700 | 78.2 | 96.2 | 92 | -4.2 |
| 3 |  | Christchurch  Ward | Work up | No | Nonin Avant 9700 | 77.5 | 96.4 | 91 | -5.4 |
| 4 |  | Christchurch Outpatients | Work up | No | Nonin Avant 9700 | 74.2 | 96.7 | 92 | -4.7 |
| 5 |  | Westmead  HDU | Heart failure | Yes | Philips Intellivue MP70** | 80 | 98 | 94 | -4 |
| 6 | Cystic fibrosis | Christchurch  Ward | Fitness to fly test | See note*** | Nonin Avant 9700 | 47.2 | 84.9 | 80 | -4.9 |
| 7 | COPD | Westmead  HDU | COPD | Yes | Philips Intellivue MP70 | 55.5 | 91.2 | 87 | -4.2 |
| 8 | COPD | Westmead Outpatients | Heart failure | No | Ohmeda Biox 3700e | 63.7 | 94.3 | 89 | -5.3 |
| 9 | COPD | Wellington Outpatients | COPD | No | Masimo Radical 7 | 74 | 95 | 91 | -4 |
| 10 | Chest wall disease | Westmead Outpatients | Chronic respiratory failure | Yes | Masimo Radical 7 | 71.6 | 95.3 | 85 | -10.3 |
| 11 | COPD | Wellington  Ward | COPD | No | Nonin 2120 | 44 | 79 | 83 | 4 |
| 12 | COPD | Wellington Outpatients | Other- not documented | No | Masimo Radical 7 | 46 | 83 | 87 | 4 |
| 13 | Neuromuscular disease | Westmead Outpatients | Neuromuscular disease | No | Welch Allyn**** | 65.8 | 94.9 | 99 | 4.1 |

COPD: Chronic Obstructive Pulmonary Disease, FiO_2_: Fraction of inspired oxygen, HDU: High dependency unit, SaO_2_: Oxygen saturation measured by arterial blood gas sample, SpO_2_: Oxygen saturation measured by standard pulse oximeter, PaO_2_: Arterial partial pressure of oxygen. Blue cells are participant SpO_2_ and SaO_2_ values which indicate oxygen therapy should be started or increased based on TSANZ Guidelines (i.e saturation value <88% in participants at risk of hypercapnia and value below 92% in participants not at risk of hypercapnia).

* At time of paired SPO_2_/SaO_2_ recording, **Monitor was used with either a GE TruSignal, Nellcor or Philips probe (specific probe not documented), ***Hypoxic gas (FiO_2_ 0.15) administered as part of a flight test, ****Welch Allyn monitor was either Welch Allyn 300 series, Welch Allyn 52000 series, Welch Allyn Spot Vital Signs LXi.

All SpO_2_ values were taken from finger probes.

Participants 8 and 13 had documented investigator concern over accuracy of the SpO_2._ In Participant 8 this was noted instability of SpO_2_ values between 89 to 95%, despite trace appearing satisfactory. In participant 13 the reason was not documented by the Investigator.

Participant 5 had peripheral vascular disease.

**Table S3 Sensitivity and specificity for SpO_2_ less than or equal to the nominated level for SaO_2_ <90%**

| **SpO_2_** | **Sensitivity** | **Specificity** |
| --- | --- | --- |
| 72 | 2.9 | 100 |
| 77 | 5.7 | 100 |
| 78 | 8.6 | 100 |
| 79 | 11.4 | 100 |
| 80 | 14.3 | 100 |
| 83 | 28.6 | 100 |
| 84 | 34.3 | 100 |
| 85 | 40.0 | 99.7 |
| 86 | 57.1 | 99.7 |
| 87 | 71.4 | 99.5 |
| 88 | 88.6 | 98.1 |
| 89 | 88.6 | 95.1 |
| 90 | 97.1 | 89.0 |
| 91 | 100 | 84.4 |
| 92 | 100 | 75.9 |
| 93 | 100 | 61.6 |
| 94 | 100 | 53.7 |
| 95 | 100 | 35.9 |
| 96 | 100 | 22.7 |
| 97 | 100 | 11.0 |
| 98 | 100 | 2.7 |
| 99 | 100 | 0.8 |
| 100 | 100 | 0.0 |

SaO_2_: Oxygen saturation measured by arterial blood gas sample, SpO_2_: Oxygen saturation measured by standard pulse oximeter (%).

**Table S4 Sensitivity and specificity for SpO_2_ less than or equal to the nominated level for PaO_2_ <60mmHg**

| **SpO_2_** | **Sensitivity** | **Specificity** |
| --- | --- | --- |
| 72 | 1.6 | 100 |
| 77 | 3.3 | 100 |
| 78 | 4.9 | 100 |
| 79 | 6.6 | 100 |
| 80 | 8.2 | 100 |
| 83 | 16.4 | 100 |
| 84 | 19.7 | 100 |
| 85 | 23.0 | 99.7 |
| 86 | 31.1 | 99.4 |
| 87 | 41.0 | 99.4 |
| 88 | 57.4 | 99.1 |
| 89 | 70.5 | 98.2 |
| 90 | 90.2 | 94.4 |
| 91 | 95.1 | 90.0 |
| 92 | 98.4 | 81.4 |
| 93 | 100 | 66.4 |
| 94 | 100 | 57.8 |
| 95 | 100 | 38.6 |
| 96 | 100 | 24.5 |
| 97 | 100 | 11.8 |
| 98 | 100 | 3.0 |
| 99 | 100 | 0.9 |
| 100 | 100 | 0.0 |

PaO_2_: Partial pressure of arterial oxygen, SpO_2_: Oxygen saturation measured by standard pulse oximeter (%).

**Figure S1 ROC curve for SpO_2_ to predict PaO_2_ <60mmHg**


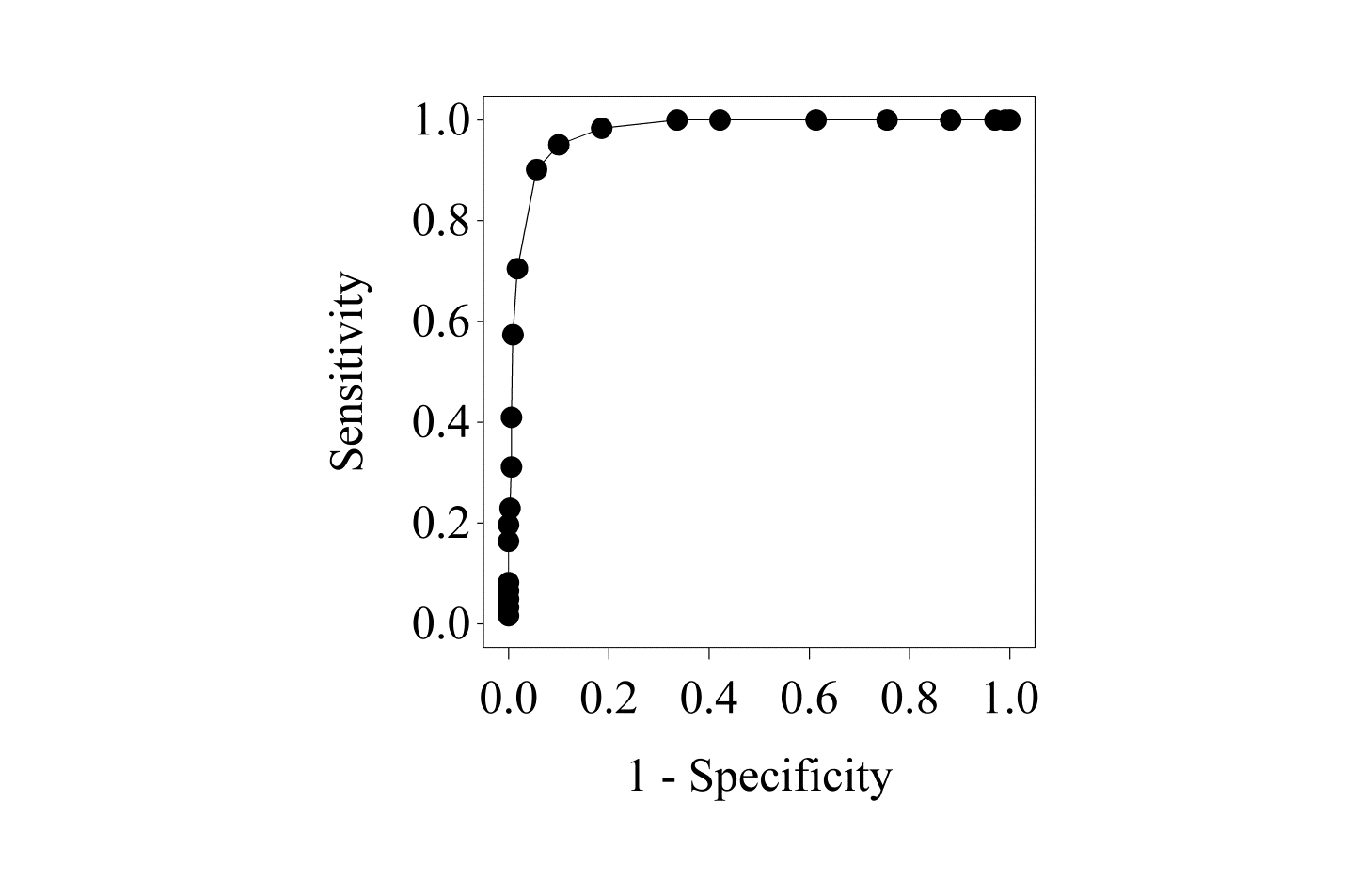


The c-statistic for the logistic regression, representing the area under the ROC curve, was 0.976.

PaO_2_: Partial pressure of arterial oxygen, SpO_2_: Oxygen saturation measured by pulse oximeter.

**Figure S2 Predicted oxygen haemoglobin dissociation curve and data from ABG samples taken in this study.**

SaO_2_


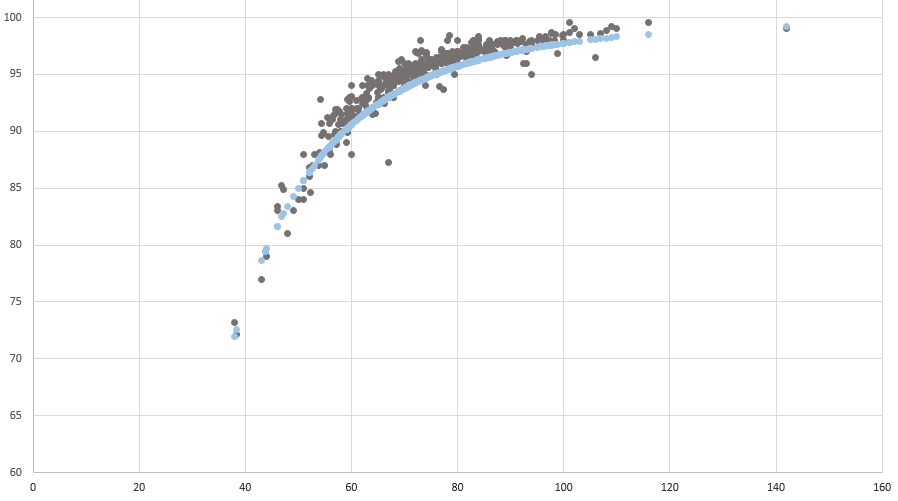


PaO_2_

ABG: Arterial blood gas, PaO_2_: Partial pressure of arterial oxygen (mmHg), SaO_2_: Oxygen saturation measured by arterial blood gas sample (%). Blue data points are predicted values calculated from PaO_2_ values obtained in this study using the simplified Severinghaus equation.^34^ Grey data points are data from participants in this study. One participant’s data omitted (PaO_2_ was 396mmHg, SaO_2_ was 100%) to retain graph axis limit formatting.

**Figure S3** Plot for bias versus SaO_2_


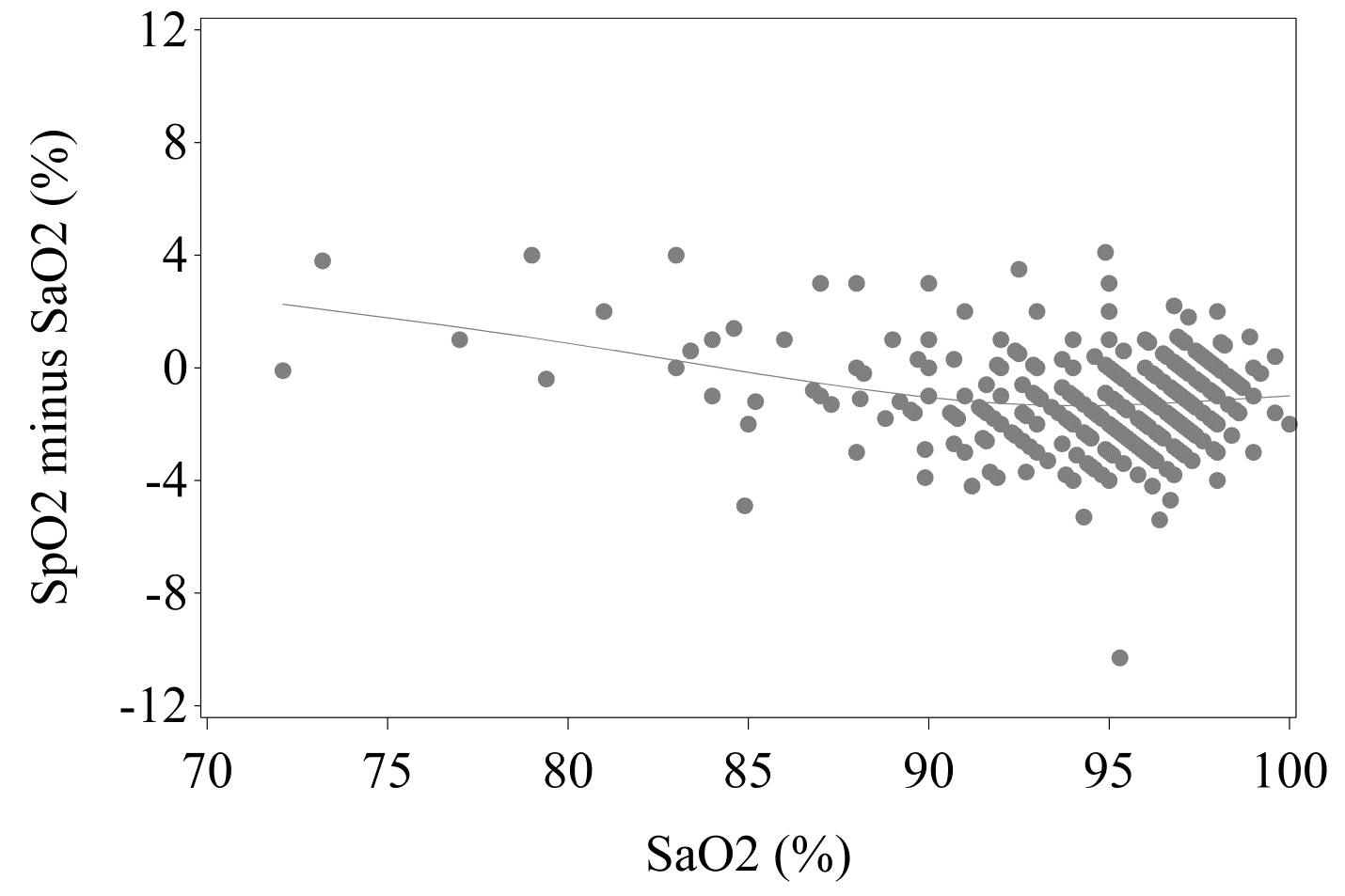


SaO_2_: Oxygen saturation measured by arterial blood gas sample, SpO_2_: Oxygen saturation measured by standard pulse oximeter.

**Table S5 Contingency Tables and ANOVA results for factors from Table 1**

| **Diabetes** | **SpO_2_ minus SaO_2_ Mean (SD)** | | |
| --- | --- | --- | --- |
| No N=320 | -1.2 (1.6) | | |
| Yes N=80 | -0.8 (1.8) | | |
| The ANOVA based F-test for evidence in difference in mean bias by Diabetes: F (1,398 DF) 3.84, P=0.05: difference -0.4 (95% CI -0.08 to 0.001). Given that the difference was not of clinical significance a Bland Altman plot was not performed. | | | |
|  | | | |
| **Location** | **SpO_2_ minus SaO_2_ Mean (SD)** | | |
| Emergency Department N=1 | -0.6 (NA) | | |
| High Dependency Unit N=18 | -1.1 (1.6) | | |
| Outpatient department N=341 | -1.2 (1.6) | | |
| Ward N=40 | -1.3 (1.6) | | |
| ANOVA based F-test for evidence in difference in mean bias: F (2,396 DF) 0.10, P=0.91. | | | |
|  | | | |
| **Position of oximeter probe** | **SpO_2_ minus SaO_2_ Mean (SD)** | | |
| Ear N=1 | -2.5 (NA) | | |
| Finger N=399 | -1.2 (1.6) | | |
| ANOVA results not reported due to result of N=1 for ear subgroup, as per methods. | | | |
|  | | | |
| **Doctor’s diagnosis of recognised condition associated with chronic respiratory failure*** | | | **SpO_2_ minus SaO_2_ Mean (SD)** |
| No N=229 | | | -1.3 (1.3) |
| Yes N=171 | | | -1.0 (1.9) |
| ANOVA based F-test for evidence in difference in mean bias: F (1,398 DF) 2.53, P=0.11  ***** Chronic obstructive pulmonary disease, obesity hypoventilation syndrome, bronchiectasis, cystic fibrosis, neuromuscular disease and chest wall deformities such as severe kyphoscoliosis | | | |
|  | | | |
| **Current tobacco smoking status** | **SpO_2_ minus SaO_2_ Mean (SD)** | | |
| Current N=43 | -1.1 (1.6) | | |
| Ex N=203 | -1.2 (1.7) | | |
| Never N=153 | -1.1 (1.5) | | |
| ANOVA based F-test for evidence in difference in mean bias: F (2,396 DF) 0.15, P=0.86. | | | |
|  | | | |
| **Fitzpatrick code** | | **SpO_2_ minus SaO_2_ Mean (SD)** | |
| Dark N=1 | | 0.1 (NA) | |
| Medium N=157 | | -1.1 (1.6) | |
| Light N=242 | | -1.2 (1.7) | |
| ANOVA based F-test for evidence in difference in mean bias: F (1,397 DF) 0.42, P=0.52. Note Dark category was excluded from analysis as N=1, as per methods. | | | |

NA: Not applicable (N=1), SaO_2_: Oxygen saturation measured by arterial blood gas sample (%), SpO_2_: Oxygen saturation measured by standard pulse oximeter (%). See Table 1 for Fitzpatrick code definitions.

**Table S6 Variation due to oximeter**

| **Oximeter** | **SpO_2_ minus SaO_2_ Mean (SD)** | **Estimates of SpO_2_ minus SaO_2_ (95% CI) by broad best linear unbiased prediction** |
| --- | --- | --- |
| Carescape Monitor B450 with a Nellcor probe | -1.5 (NA) | -1.2 (-2.3 to -0.03) |
| GE Dash 3000 | -2 (0) | -1.2 (-2.3 to -0.2) |
| Masimo Radical 7 | -0.5 (1.9) | -0.6 (-0.9 to -0.3) |
| Masimoset Quartz Unspecified* | -0.5 (NA) | -1.1 (-2.2 to 0.03) |
| Masimoset Quartz Q400 | -1.0 (1.3) | -1.1 (-1.7 to -0.5) |
| Nonin 2120 | -0.4 (2.3) | -0.9 (-1.7 to -0.03) |
| Nonin 2140 | -2 (NA) | -1.2 (-2.3 to -0.1) |
| Nonin Avant Unspecified* | -2.4 (NA) | -1.2 (-2.3 to -0.1) |
| Nonin Avant 4000 | -1.5 (1.2) | -1.5 (-1.8 to -1.1) |
| Nonin Avant 9700 | -1.4 (1.4) | -1.4 (-1.7 to -1.1) |
| Nonin Lifesense Medair | -1.5 (1.5) | -1.4 (-1.9 to -1.0) |
| Novametrix Model 512 | 0 (NA) | -1.1 (-2.2 to 0.1) |
| Ohmeda Biox 3700E with a GE TruSignal or Nellcor probe | -2.0 (2.0) | -1.4 (-2.3 to -0.5) |
| Philips Intellivue MP70 with a GE TruSignal , Nellcor or Philips probe | -1.1 (1.6) | -1.1 (-1.8 to -0.5) |
| Welch Allyn** with a Nellcor probe | -0.3 (2.0) | -0.7 (-1.3 to -0.04) |

The machine by machine estimates are all very similar, consistent with the high value of the intra-class correlation coefficient. NA: Not applicable (N=1), SaO_2_: Oxygen saturation measured by arterial blood gas sample (%), SpO_2_: Oxygen saturation measured by standard pulse oximeter (%). *Unspecified by investigator. **Welch Allyn monitors were either Welch Allyn 300 series, Welch Allyn 52000 series, Welch Allyn Spot Vital Signs LXi.
